# Comparative Evaluation of Polymeric Nanocarriers for DNA Vaccine Delivery Against *Avian Orthoavulavirus 1* in Chickens

**DOI:** 10.3390/v18050581

**Published:** 2026-05-21

**Authors:** Ahmed H. Khattab, Mahmoud Bayoumi, Zienab E. Eldin, Basem M. Ahmed, Haitham M. Amer

**Affiliations:** 1 Virology Department, Faculty of Veterinary Medicine, Cairo University, Giza 12211, Egypt; a.khattab@cu.edu.eg (A.H.K.); mahmoud.bayoumi@cu.edu.eg (M.B.); basem-ahmed@cu.edu.eg (B.M.A.); 2 Host Pathogen Interactions (HPI) and Disease Intervention & Prevention (DIP) Programs, Texas Biomedical Research Institute, San Antonio, TX 78227, USA; 3 Materials Science and Nanotechnology Department, Faculty of Postgraduate Studies for Advanced Sciences, Beni-Suef University, Beni-Suef 62511, Egypt; t-zienabessam@zewailcity.edu.eg; 4 Faculty of Veterinary Medicine, Egyptian Chinese University, Cairo 11734, Egypt

**Keywords:** Newcastle disease (ND), *Avian Orthoavulavirus 1* (AOAV-1), DNA vaccine, nanoparticles-based delivery, polymeric nanoparticles, chitosan, PLGA, PAMAM-dendrimers

## Abstract

Vaccination represents the cornerstone of Newcastle disease control. Nanotechnology offers a promising approach to improve the effectiveness of DNA vaccines, supporting their use as an alternative to conventional platforms. Herein, the *Avian Orthoavulavirus 1* (AOAV-1) *fusion* (*F*) gene was cloned into a DNA expression plasmid (pDNA). After validating the constructed pDNA-F and confirming robust intracellular protein expression in vitro, three polymeric nanoparticles (NPs)-based formulations were generated using Chitosan (Cs), poly(lactic-co-glycolic) (PLGA), and poly(amidoamine) (PAMAM)-Dendrimers. Physicochemical characterization, stability assessment, and in vitro release analysis confirmed nanoparticle formation and effective DNA incorporation. In vivo experiments were conducted to comparatively evaluate the immunogenicity, particularly the immune priming capacity, and protective efficacy of nanoparticle-based formulations and naked pDNA-F, all tested in parallel at standardized pDNA doses via intranasal (IN) and intramuscular routes. PAMAM-Dendrimers-pDNA-F IM group demonstrated superior efficacy, with 100% survival, the highest post-challenge anamnestic antibody titers, and a pronounced reduction in viral RNA shedding. PLGA-NPs-pDNA-F IN group demonstrated enhanced efficacy, with 90% survival. Naked pDNA-F surpassed the Cs-NPs-pDNA-F in both immune priming and clinical protection, with Cs-NPs-pDNA-F exhibiting the lowest overall performance. These findings highlight that DNA vaccine performance depends on both carrier type and administration route, with PAMAM dendrimers and PLGA enhancing efficacy, whereas chitosan demonstrated reduced efficacy under the tested conditions.

## 1. Introduction

The poultry industry strongly supports global food security, a central pillar of the One Health framework [1]. However, infectious viral diseases continue to threaten the sustainability of poultry production. Newcastle disease (ND) remains one of the most devastating viral diseases affecting both domestic and wild birds, causing significant economic losses and negatively impacting poultry production and international trade; moreover, it has a zoonotic potential [2,3,4,5]. The high transmissibility of ND is driven by viral replication at mucosal surfaces. Both respiratory and digestive mucosal tissues serve as primary sites of viral entry and initial replication, facilitating local infection and subsequent systemic dissemination [2,3]. Accordingly, immunity at these mucosal surfaces is critical not only for mitigating disease severity but also for limiting viral replication and subsequent shedding, thereby reducing transmission within poultry flocks. These considerations highlight the importance of mucosal vaccination [6,7]. ND is caused by *Avian Orthoavulavirus 1* (AOAV-1), a member of the Paramyxoviridae family currently assigned by the ICTV to the species *Orthoavulavirus javaense* (OAV-j), formerly known as Newcastle disease virus (NDV). AOAV-1 is an enveloped virus with a non-segmented, negative-sense, single-stranded RNA genome that encodes six structural proteins: nucleoprotein [N], phosphoprotein [P], matrix protein [M], fusion protein [F], hemagglutinin-neuraminidase [HN], and large polymerase protein [L], and two non-structural proteins (V and W) [8,9]. HN and F are the major envelope glycoproteins that play critical roles in viral-cell interactions and in determining strain virulence [10]. F protein is a type 1 glycoprotein responsible for membrane fusion after receptor binding is mediated by AOAV-1 HN protein [11]. Its functional domains, particularly the fusion peptide and heptad repeats, are the mechanical engines driving the transition from pre- to post-fusion state [10,12]. Antibodies targeting the F protein neutralize the virus by physically blocking these domains, thereby preventing membrane fusion and genome delivery into the host cell. Consequently, an effective anti-F immune response is the most critical factor in mitigating mortality and suppressing viral shedding [13].

Alongside strict biosecurity measures, vaccination represents the cornerstone of effective control against ND. Traditional live and inactivated vaccines are widely used in the commercial poultry sector; however, several limitations compromise their availability and field effectiveness, particularly in low and middle-income countries. These constraints include, but are not limited to, production and supply challenges, high costs, and limited adaptability to the rapid evolution of circulating strains, thereby increasing the likelihood of antigenic mismatch between vaccine strains and currently circulating virulent genotypes [14,15,16,17,18,19,20]. Additionally, maternally derived antibodies (MDA) may interfere with early vaccine responses in young chicks, thereby contributing to incomplete protection and potential vaccination failure [21]. Molecular-based vaccines, particularly DNA vaccines, offer a promising alternative to the conventional vaccination platforms. DNA vaccines are developed by inserting a gene encoding immunodominant antigen(s) into a eukaryotic expression plasmid (pDNA). Upon delivery into host cells, the encoded antigen is de novo expressed intracellularly, allowing for correct protein folding, natural post-translational modifications, and efficient processing and presentation via both major histocompatibility complex pathways. In addition to their antigen expression characteristics, DNA vaccines exhibit a distinct immunological profile characterized by efficient induction of both innate and adaptive immune responses. Unmethylated CpG motifs within the plasmid backbone act as intrinsic immunostimulatory elements, activating avian Toll-like receptor 21 (TLR21) and initiating innate immune signaling that promotes a Th1-biased cytokine environment, including interferon-γ and interleukin-2. This innate activation drives the development of cell-mediated immunity, as endogenous antigen expression facilitates efficient presentation via MHC class I pathways, leading to activation of antigen-specific CD8^+^ cytotoxic T lymphocytes, which are critical for controlling intracellular viral infections. Importantly, DNA vaccines function as effective priming platforms, establishing antigen-specific memory T and B cells that enable rapid and enhanced anamnestic responses upon subsequent antigen exposure [22,23,24,25,26]. DNA vaccines also provide practical advantages, including favorable safety profiles, relatively low production costs, and the use of established manufacturing platforms that can be rapidly adapted to emerging variants. Additionally, the final product is relatively stable, facilitating storage and distribution without stringent cold-chain requirements [22,23,24,25].

Despite these advantages, several obstacles hinder the clinical translation and widespread commercial application of DNA vaccines in the poultry industry. The immunogenicity and protective efficacy of naked pDNA are often suboptimal, primarily due to inefficient cellular uptake and low antigen expression levels [26]. Furthermore, conventional intramuscular administration mainly induces systemic immune responses. It is also generally insufficient to elicit robust mucosal immunity [6,7]. These limitations highlight the need for strategies that enhance DNA vaccine delivery and biological performance, shifting research efforts toward the development of more effective delivery systems that improve cellular uptake, antigen expression, and mucosal immune activation [27].

Advances in nanotechnology have provided promising platforms for the efficient delivery of DNA vaccines. Nanoparticle-based carriers can protect pDNA from premature extracellular degradation, enhance cellular uptake, and improve intracellular delivery, thereby increasing antigen expression [28,29]. These improvements are often reflected in enhanced immune priming and in vivo protective efficacy [28,29]. A wide range of polymeric nanoparticle delivery systems has been explored for DNA vaccine applications. Among these, natural linear polymers, such as chitosan, synthetic linear polymers, such as poly(lactic-co-glycolic) acid (PLGA), and branched polymeric architectures, such as poly(amidoamine) (PAMAM) dendrimers, have attracted scientific attention. Each of these platforms offers distinct advantages in DNA concentration, protection, cellular interactions, and intracellular trafficking, which are critical determinants of immune priming and overall vaccine efficacy [30,31,32].

Therefore, various polymeric nanoparticles were used in this study to formulate pDNA-F vaccines, which demonstrated variable efficacy compared with the naked pDNA-F vaccine. PAMAM-Dendrimers nanoparticles demonstrated superior efficacy following intramuscular administration, while PLGA nanoparticles yielded the most favorable results when administered intranasally. Naked pDNA-F surpassed the chitosan-pDNA-F formulation in both immune priming and clinical protection. These results highlight the significance of route-dependent formulation performance and indicate that nanotechnology can both improve and impair the effectiveness of DNA vaccines.

## 2. Materials and Methods

### 2.1. Cells and Viruses

Chicken fibroblast (DF-1) cells were maintained in Dulbecco’s modified Eagle medium (DMEM) supplemented with 5% fetal bovine serum (FBS) and 1× antibiotic-antimycotic solution (Thermo Fisher Scientific, Waltham, MA, USA) at 37 °C in a humidified 5% CO_2_ incubator. The Egyptian AOAV-1 isolate (Genbank accession no: F19753D) served as the template for *F*-gene amplification to generate the transgene and was subsequently used as a challenge virus in the animal experiment. The titer of the challenge virus was determined in 9–11-day-old specific-pathogen-free (SPF) embryonated chicken eggs. Briefly, ten-fold serial dilutions of the virus stock were inoculated via the allantoic cavity, and embryo mortality was recorded daily for 5 days. The virus titer was calculated using the Reed and Muench method and expressed as the 50% egg infectious dose (EID_50_) per 0.2 mL [33]. The challenge dose used in the animal experiment was standardized to 10^5.5^ EID_50_ per bird [34].

### 2.2. Construction of AOAV-1 F Gene Expression Cassette

Viral RNA was extracted from virus-containing allantoic fluid using QIAamp Viral RNA Mini Kit (Qiagen, Germantown, MD, USA) according to the manufacturer’s protocol. First-strand cDNA synthesis was carried out using RevertAid First Strand cDNA Synthesis Kit (ThermoFisher Scientific, Waltham, MA, USA) and random hexamer primers. The full-length *F*- gene of AOAV-1 was amplified using gene-specific primers modified to include SacI and SmaI restriction sites (MB-OAV-j-F: 5′-TTCGAGCTCGCCACCATGGGCTCCAAACCTTCTACC-3′ and MB-OAV-j-R: 5′-TCCCCCGGGTGCTCTCGTGGTGGCTCTC-3′). PCR amplification was performed with Phusion High-Fidelity Master Mix (Thermo Fisher Scientific) according to the manufacturer’s guidelines. Thermal cycling conditions included an initial denaturation at 95 °C for 5 min, 35 cycles of 95 °C for 10 s, 54 °C for 30 s, and 72 °C for 120 s, and a final extension at 72 °C for 10 min. The product was visualized on 1% agarose gel, and the specific band was excised and purified using the GeneJET Gel Extraction Kit (Thermo Fisher Scientific). The concentration and purity of the purified product were measured on a NanoDropTM spectrophotometer (Thermo Fisher Scientific).

The purified PCR product of the *F* gene was digested with SacI and SmaI and inserted into the pCAGGS-FLAG-COOH expression plasmid. The pCAGGS vector is driven by the CAG promoter complex, which combines the chicken β-actin promoter with a cytomegalovirus (CMV) enhancer to facilitate robust ectopic expression [35]. Successful cloning was first verified by restriction digestion with the same enzymes and colony PCR using gene-specific primers. In addition, Sanger sequencing with gene-specific primers confirmed successful integration of the *F* gene. To further validate the entire construct, the recombinant plasmid was analyzed by next-generation sequencing (NGS).

### 2.3. In Vitro Expression of the Recombinant F Protein

#### 2.3.1. Indirect Immunofluorescent Assay

DF-1 cells were seeded onto sterile glass coverslips in 24-well tissue culture plates and incubated overnight at 37 °C in a humid chamber. Cells at 70–90% confluency were transfected with the pDNA-F (500 ng per well) with Lipofectamine 3000™ (Thermo Fisher Scientific), according to the manufacturer’s guidelines. Control wells were transfected with the empty pCAGGS vector. After incubation at 37 °C for 24 h, cells were fixed with 4% paraformaldehyde for 1 h and permeabilized with 0.1% Triton X-100 for 10 min. To block non-specific binding, samples were treated with 0.5% bovine serum albumin (BSA) for 1 h at room temperature. Coverslips were then incubated with rabbit anti-FLAG primary antibody (1:1000) for 1 h at 37 °C, followed by three PBS washes, then incubated with Alexa Fluor 488–conjugated anti-rabbit IgG secondary antibody (1:2000; Invitrogen, Carlsbad, CA, USA) for 1 h at 37 °C. After three additional PBS washes, coverslips were mounted in 50% glycerol medium. Fluorescence was observed under an inverted Olympus IX81 microscope (Olympus America Inc., Centre Valley, PA, USA) equipped with Alexa Fluor 488 excitation and emission filters.

#### 2.3.2. Western Immunoblotting

DF-1 cells were seeded in 6-well tissue culture plates and similarly transfected with pDNA-F (2000 ng per well) using Lipofectamine 3000™ (Thermo Fisher Scientific), according to the manufacturer’s guidelines. After 48 h, cells were washed twice with ice-cold PBS, detached with trypsin, and pelleted by centrifugation at 10,000× *g* for 30 min at 4 °C. Pellets were resuspended and lysed on ice for 30 min in NP-40 lysis buffer (Thermo Fisher Scientific) containing protease inhibitors (Thermo Fisher Scientific), and then clarified by centrifugation at 18,600× *g* for 15 min at 4 °C. Protein concentrations were determined, and equal amounts of lysate were mixed with Laemmli loading buffer, denatured by heating, and separated by SDS-PAGE. Proteins were transferred to a nitrocellulose membrane by wet transfer and blocked with 5% non-fat dry milk in PBS-Tween (PBST) for 1 h at room temperature. Membranes were incubated overnight at 4 °C with rabbit anti-FLAG primary antibody (1:1000), washed three times in PBST, and incubated for 2 h at room temperature with HRP-conjugated anti-rabbit IgG secondary antibody (1:2000). To confirm equal loading, membranes were reprobed with mouse anti-β-actin primary antibody followed by HRP-conjugated anti-mouse IgG. Positive reactions were developed with a chemiluminescent substrate (Thermo Fisher Scientific) and visualized using a ChemiDoc imaging system (Bio-Rad, Hercules, CA, USA).

### 2.4. Scaling-Up and Purification of pDNA-F

Following confirmation of cloning and in vitro expression, pDNA-F was amplified in TOP10 *E. coli* (Thermo Fisher Scientific) and then purified using QIAGEN^®^ Plasmid Maxi Kit (Qiagen) according to the manufacturer’s instructions. Plasmid concentration and purity were assessed using NanoDrop™ spectrophotometer, and only preparations with A_260_/A_280_ ratios of 1.8–2.0 were used. The purified endotoxin-free pDNA-F was employed for nanoparticle-based formulations and in vivo immunization studies.

### 2.5. Preparation of Nanoparticle-Based DNA Vaccine Formulations

#### 2.5.1. Preparation of Chitosan Nanoparticles (Cs NPs)—Based pDNA-F Vaccine

Cs NPs loaded with pDNA-F (Cs-NPs-pDNA-F) were prepared using the ionic gelation method under mild conditions. A 0.3% (*w*/*v*) chitosan solution was prepared by dissolving medium molecular weight chitosan (75% degree of deacetylation; Sigma-Aldrich, St. Louis, MO, USA) in 1% (*v*/*v*) analytical-grade acetic acid (Sigma-Aldrich) and stirring overnight at room temperature (25 ± 0.5 °C) until complete dissolution. The pH of the solution was adjusted to 5.5 using 1N sodium hydroxide (Sigma-Aldrich) to optimize interaction with pDNA-F. Separately, pDNA-F was diluted to a concentration of 0.5 µg/mL using PBS (pH 7.4; Thermo Fisher Scientific). Nanoparticles were formed by mixing equal volumes of Cs solution and pDNA-F solution at an optimized N/P ratio of 3:1 (see Supplementary Methods S1 for full calculation details). The mixture was stirred continuously for 1 h at room temperature (25 ± 0.5 °C) to facilitate interactions between Cs and DNA. To ensure uniform nanoparticle formation, the solution was vortexed for 30 s at 2500 rpm. The nanoparticle suspension was centrifuged at 18,600× *g* for 15 min at 4 °C, and the pellet was washed three times with PBS (pH 7.4) to remove unbound DNA and impurities [36,37]. The prepared nanoparticles were freeze-dried and stored at 4 °C until use.

#### 2.5.2. Preparation of PLGA Nanoparticles (PLGA NPs)-Based pDNA-F Vaccine

PLGA NPs loaded with pDNA-F (PLGA-NPs-pDNA-F) were prepared using the double emulsion-solvent evaporation method [38]. First, an organic phase was prepared by dissolving PLGA (50:50, 40–75 kDa; Sigma-Aldrich) at a concentration of 30 mg/mL in dichloromethane (DCM; Thermo Fisher Scientific). Separately, the aqueous phase containing pDNA-F was prepared at a concentration of 0.5 µg/mL in deionized water. To form the primary emulsion (water-in-oil), 2 mL of the aqueous phase was added to 4 mL of the organic phase and sonicated using a probe sonicator at 50 W for 30 s. The primary emulsion was then added dropwise to 10 mL of a 2% (*w*/*v*) poly(vinyl alcohol) solution (PVA, 30–70 kDa; Thermo Fisher Scientific) to form a double emulsion (water-in-oil-in-water). This mixture was sonicated again at 50 W for 60 s to stabilize the emulsion. The resulting double emulsion was stirred at room temperature for 6 h to allow complete evaporation of DCM, resulting in the formation of PLGA NPs. The nanoparticles were collected by centrifugation at 21,400× *g* for 15 min at 4 °C. The pellet was washed three times with deionized water to remove residual PVA and unencapsulated pDNA-F. The purified nanoparticles were lyophilized and stored at −20 °C until use [39].

#### 2.5.3. Preparation of PAMAM Dendrimers–Based pDNA-F Vaccine

PAMAM G5 dendrimers-loaded pDNA-F (PAMAM-Dendrimers-pDNA-F) were prepared using a self-assembly process based on electrostatic interactions between the cationic dendrimers and the anionic pDNA backbone. A stock solution of generation 5 PAMAM dendrimers (molecular weight: 28,826 Da, diameter: 5.4 nm, 128 surface amine groups; Sigma-Aldrich, St. Louis, MO, USA, Cat. No. 412368) was prepared by diluting the commercial 5% (*w*/*v*) methanolic solution 1:10 (*v*/*v*) in sterile 20 mM HEPES buffer (pH 7.4, filtered through 0.22 µm PES membrane) and subsequent removal of methanol by rotary evaporation at 40 °C under reduced pressure (100 mbar) for 30 min, followed by gentle nitrogen gas flushing for 10 min to ensure complete organic solvent elimination. The final dendrimer concentration was adjusted to 1.0 mg/mL (34.7 µM) in HEPES buffer, and the solution was stored at 4 °C for no longer than 2 weeks prior to use. The pDNA-F was diluted to 100 µg/mL in 10 mM HEPES buffer (pH 7.4, A260/A280 ratio 1.80–2.00). Nanoparticle formation was initiated by adding the PAMAM G5 dendrimers solution dropwise (10 µL per 30 s interval) to an equal volume of pDNA solution under gentle vortex mixing at 400 rpm using a Vortex-Genie 2 mixer (Scientific Industries, Bohemia, NY, USA) to achieve a final nitrogen-to-phosphate (N/P) ratio of 10:1 (see Supplementary Methods S1 for full calculation details). Following the addition of the dendrimer solution, the mixture was immediately vortexed for 30 s at maximum speed, then incubated at room temperature (25 ± 1 °C) without agitation for 30 min to allow complete electrostatic self-assembly and formation of stable, compact nanoparticles. The resulting nanoparticle suspension was then centrifuged at 1200× *g* for 5 min at 4 °C to remove any large aggregates, and the supernatant was collected. For all subsequent experiments, the final pDNA concentration within the nanoparticle formulation was adjusted to 50 µg/mL using 10 mM HEPES buffer (pH 7.4).

### 2.6. Physicochemical Characterization of Nanoparticles

#### 2.6.1. Hydrodynamic Size, Polydispersity Index, and Zeta Potential Analysis

The hydrodynamic diameter, polydispersity index (PDI), and zeta potential of the nanoparticles were determined using Zetasizer Nano ZS (Malvern, UK) at 25 °C. Samples were dispersed in deionized water prior to analysis. Therefore, the measurements reflected the hydrodynamic behavior of the suspended particle population. Measurements were performed in triplicate, and the obtained data were used to evaluate particle size distribution, homogeneity, and surface charge characteristics.

#### 2.6.2. Scanning Electron Microscopy (SEM)

The surface morphology of nanoparticles was examined using SEM. Samples were analyzed using Philips XL30 SEM (Philips, Amsterdam, The Netherlands) to evaluate particle shape, surface topography, and morphological characteristics. The obtained images represent particle morphology after sample preparation and drying.

#### 2.6.3. Fourier Transform Infrared (FTIR) Spectroscopy

Fourier-transform infrared (FTIR) spectra were recorded in the range of 4000–400 cm^−1^ using Vertex 70 FTIR spectrometer (Bruker, Mannheim, Germany). Samples were prepared as potassium bromide pellets prior to analysis. The spectra were acquired at a resolution of 1 cm^−1^, and each spectrum represented the average of three consecutive scans. FTIR analysis was performed to identify the characteristic functional groups and to confirm molecular interactions within the samples.

#### 2.6.4. Entrapment Efficiency (EE%) and Loading Capacity (LC%)

The entrapment efficiency (EE%) and loading capacity (LC%) of nanoparticles loaded with pDNA were determined using NanoDrop spectrophotometry. Briefly, 1 mg of nanoparticles was dispersed in 100 µL of 0.1 N HCl to extract the nanoparticle-associated DNA for spectrophotometric quantification. The suspension was vortexed briefly and centrifuged at 21,400× *g* for 15 min to remove insoluble materials. A 1 µL aliquot of the supernatant was analyzed using a NanoDrop spectrophotometer, and the absorbance was measured at 260 nm. DNA concentration was automatically calculated by the NanoDrop One Software v1.2 based on the Beer–Lambert law. To determine entrapment efficiency, the concentration of free (non-encapsulated) pDNA-F in the supernatant collected during nanoparticle preparation was measured separately. The EE% and LC% were calculated using the following equations:

EE% = [(Total pDNA added − Free pDNA)/Total p.DNA added] × 100(1)

LC% = [Encapsulated pDNA/Weight of nanoparticles] × 100(2)

All measurements were performed in triplicate to ensure accuracy and reproducibility.

### 2.7. In Vitro DNA Release Study

The in vitro release profile of pDNA-F from the prepared nanoparticles was evaluated under both baseline and biologically relevant conditions. Briefly, 10 mg of pDNA-F–loaded nanoparticles were dispersed in 1 mL of release medium and incubated at 37 °C with gentle agitation (100 rpm). Three release media were used: (i) PBS (pH 7.4), (ii) PBS containing 0.5% (*w*/*v*) porcine stomach mucin (Type II) to simulate the mucosal environment, and (iii) PBS containing 10% (*v*/*v*) fetal bovine serum (FBS) to simulate a protein-rich biological milieu. At predetermined time intervals (0, 0.5, 1, 2, 4, 8, 12, 24, 48, and 72 h), samples were centrifuged at 21,400× *g* for 15 min to sediment the nanoparticles. A 100 µL aliquot of the supernatant was carefully collected and immediately replaced with an equal volume of fresh, pre-warmed medium to maintain sink conditions. Collected supernatants were stored at 4 °C until analysis. The amount of released pDNA-F was quantified using a NanoDrop™ spectrophotometer by measuring absorbance at 260 nm. A 1 µL aliquot of each sample was loaded onto the NanoDrop pedestal, and DNA concentration was automatically calculated using the Beer–Lambert equation. The cumulative percentage of DNA release was calculated according to Equation (3):

(3) $$\mathrm{Cumulative}\;\mathrm{release}\;(\%)\;=\left(\frac{\sum_{i}^{n}=1mi}{m\left(encapsulated\right)}\right)\times 100$$

where m_i_ represents the mass of pDNA-F released at each sampling time point (corrected for dilution), and m(encapsulated) denotes the total amount of DNA initially entrapped within the nanoparticles. All experiments were performed in triplicate.

### 2.8. In Vitro Stability Study

The stability of pDNA-F–loaded nanoparticles was further evaluated under nuclease-containing and protein-containing conditions to better reflect biologically relevant extracellular environments. For the DNase I protection assay, nanoparticle formulations containing an equivalent amount of pDNA-F were incubated in PBS supplemented with 1 U/mL DNase I and 5 mM MgCl_2_ at 37 °C for 30 and 60 min. Naked pDNA-F incubated under the same conditions served as a control. After incubation, DNase I activity was terminated by adding EDTA to a final concentration of 25 mM, followed by heating at 65 °C for 10 min. The pDNA-F was then recovered from the formulations using the respective dissociation/extraction procedure and analyzed by 1% agarose gel electrophoresis to assess the integrity of the protected DNA.

For the serum stability assay, nanoparticle formulations were incubated in PBS containing 10% (*v*/*v*) fetal bovine serum (FBS) at 37 °C under gentle agitation. Samples were collected at 0.5, 1, 2, 4, 8, 24, and 48 h and analyzed for residual intact pDNA-F and formulation integrity. Released or recovered DNA was assessed spectrophotometrically, while formulation stability was further interpreted based on changes in release behavior relative to PBS. All experiments were performed in triplicate.

### 2.9. In Vivo Evaluation of DNA Vaccine Formulations

#### 2.9.1. Experimental Animal Groups and Vaccination Protocol

One-day-old broiler chicks were maintained in controlled experimental facilities until 11 days of age. At that time, birds were randomly divided into 10 groups (10 birds each). Eight groups were immunized with one of the four pDNA-F-Nanoparticle formulations, including Cs-NPs-pDNA-F, PLGA-NPs-pDNA-F, PAMAM-Dendrimers-pDNA-F, and Naked pDNA-F (delivered either intranasally (IN, 4 groups) or intramuscularly (IM, 4 groups). The two remaining groups served as controls: non-vaccinated and challenged group (Group 1), while the other remained non-vaccinated and unchallenged (Group 2; Table 1). DNA vaccination was performed twice, at 11 and 21 days of age, with each bird receiving 40 µg of pDNA-F per dose. Due to formulation-dependent differences in DNA loading capacity, the final administration volume varied slightly among vaccine groups, with an approximate volume of 100 µL per bird. Accordingly, the total nanoparticle-carrier mass delivered per bird was not equivalent across groups.

For intranasal immunization, vaccines were administered bilaterally using a calibrated micropipette with low-retention sterile filter tips, while maintaining continuous mixing to ensure formulation homogeneity. Birds were manually restrained in a horizontal position, and the beak was held closed briefly after administration to facilitate inhalation and minimize dose loss. For intramuscular immunization, vaccines were injected into the pectoral muscle using sterile 23-gauge needles under gentle manual restraint.

#### 2.9.2. Serological Evaluation of Humoral Immunity

To evaluate humoral immune responses, Serum samples were collected on days 1 and 11 of age to assess maternally derived antibody (MDA) levels and their decline. Sera collected on days 21, 28, and 35 were used to evaluate post-vaccination antibody responses. Sera collected on day 42 were used to assess post-challenge antibody titers. At each time point, serum samples were collected from five birds randomly selected from each group, with no fixed individual tracking across time points. Antibodies against AOAV-1 F protein were quantified using an NDV-F indirect ELISA kit (BioChek BV, Reeuwijk, The Netherlands), according to the manufacturer’s instructions, with a cut-off value of ≥0.3 considered positive.

#### 2.9.3. Evaluation of Protective Efficacy

On day 35, all groups except group 10 were intranasally challenged with 0.2 mL of a virulent AOAV-1 genotype VII Egyptian strain at a dose of 10^5.5^ EID_50_/0.2 mL. Birds were monitored twice daily for clinical signs and mortality. Cloacal swabs were collected on days 3, 5, and 7 post-challenge to assess viral shedding using RT-qPCR targeting the *F* gene (HERA RT-qPCR kit with custom primers and probe, according to the manufacturer’s instructions).

### 2.10. Statistical Analysis

Unpaired Student’s T-test was used to compare the IN and IM groups for each preparation. One-way analysis of variance (ANOVA) followed by Dunnett’s multiple comparison test was applied in multiple group comparisons for a single factor. *p*-values were calculated using GraphPad Prism 8 (GraphPad Software, San Diego, CA, USA; www.graphpad.com). Data are presented as mean ± standard deviation (SD). In the in vivo experiments, the experimental unit was the individual bird, with 5 birds randomly sampled per group at each time point (*n* = 5) from a total of 10 birds per group. Survival data were analyzed using the Kaplan–Meier method, and differences between groups were evaluated using the log-rank (Mantel–Cox) test. Statistical significance was denoted as follows: ns (*p* > 0.05), * *p* ≤ 0.05, ** *p* ≤ 0.01, *** *p* ≤ 0.001, **** *p* ≤ 0.0001.

## 3. Results

### 3.1. Generation of pDNA-F and Characterization of the Recombinant F Protein

**Figure 1:**
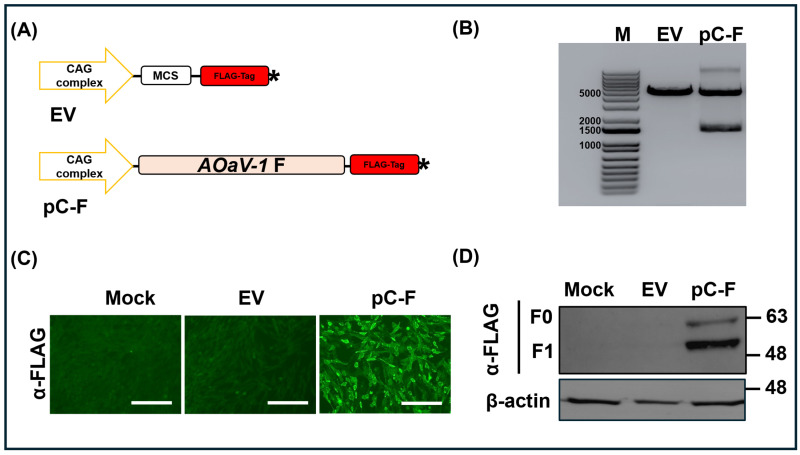
Generation and characterization of the Recombinant AOAV-1 F protein. (**A**) Schematic representation of the recombinant pCAGGS- AOAV-1 F-FLAG-COOH plasmid is driven by the CAG promoter complex (yellow arrow), which combines the chicken β-actin promoter with a cytomegalovirus (CMV) enhancer (pC-F). and the empty vector (EV). The FLAG tag showed as a red box, and the stop codon is represented by an asterisk. (**B**) Gel electrophoresis analyses of the vectors represented at (**A**), showing the restriction digestion pattern after double digestion using SacI and SmaI restriction. (**C**) Ectopic cytoplasmic expression of pC-F in chicken fibroblast DF1 transfected cells, in comparison to mock-transfected and EV-transfected cells using indirect immunofluorescence assay. Scale bars are 300 µm. (**D**) Western blot-based validation of expression of pC-F in DF1. EV- and Mock-transfected cells were included. β-actin was also included as a loading control. Molecular weight markers are indicated on the right.

Full-length *fusion (F)* gene of the Egyptian *AOAV-1* was amplified using gene-specific primers modified to include SacI and SmaI restriction sites to insert it into pCAGGS-FLAG-COOH expression plasmid (Figure 1A). Double enzymatic digestion of pDNA-F using SacI and SmaI generated two fragments of the expected sizes—approximately 4.7 kb for the pCAGGS-FLAG-COOH backbone and 1.7 kb for the full-length F insert (Figure 1B). In contrast, the empty plasmid showed only a single band. Colony PCR using gene-specific primers also generated the expected full-length F amplicon (~1.7 kb) in all positive clones. Complete plasmid sequencing using NGS further verified that the *F* gene was inserted in the correct orientation and was free of mutations, confirming full integrity of the recombinant construct.

Chicken DF1 cell line transfected with pDNA-F exhibited strong cytoplasmic green fluorescence. In contrast, mock-transfected cells and cells transfected with empty pCAGGS control showed negligible background fluorescence (Figure 1C), confirming successful expression of the recombinant FLAG-tagged-F protein. Western blot analysis demonstrated robust expression of the FLAG-tagged F protein, revealing bands at approximately 63 kDa (F0 precursor) and 48 kDa (F1 cleavage product) in pDNA-F-transfected cell lysates. As expected, specific bands were not detected in either the empty vector or the non-transfected control cells. β-actin (~42 kDa) was readily detected in all samples, confirming equal protein loading (Figure 1D). These findings collectively indicated successful construction and expression of the recombinant pDNA-F plasmid.

### 3.2. Physicochemical Characterization of the pDNA-F-Loaded Nanoparticles

#### 3.2.1. Hydrodynamic Size, Polydispersity Index (PDI), and Zeta Potential

The physicochemical properties of pDNA-F-loaded nanoparticles are summarized in Table 2. Dynamic light scattering analysis revealed significant differences in particle size among the three formulations. PAMAM-Dendrimers-pDNA-F exhibited the smallest mean hydrodynamic diameter, followed by Cs-NPs-pDNA-F, while PLGA-NPs-pDNA-F showed the largest particle size. Moreover, all formulations demonstrated relatively narrow size distributions, as indicated by low PDI values. PAMAM-Dendrimers-pDNA-F showed the lowest PDI, indicating the highest degree of homogeneity, followed by Cs-NPs-pDNA-F and PLGA-NPs-pDNA-F. Furthermore, zeta potential measurements revealed distinct surface charge characteristics among the formulations. Both PAMAM-Dendrimers-pDNA-F and Cs-NPs-pDNA-F exhibited positive surface charges. In contrast, PLGA-NPs-pDNA-F displayed a negative zeta potential value (Table 2). These differences are consistent with the intrinsic chemical properties of the carrier materials and may influence nanoparticle stability and cellular interaction.

#### 3.2.2. Scanning Electron Microscopy (SEM)

**Figure 2:**
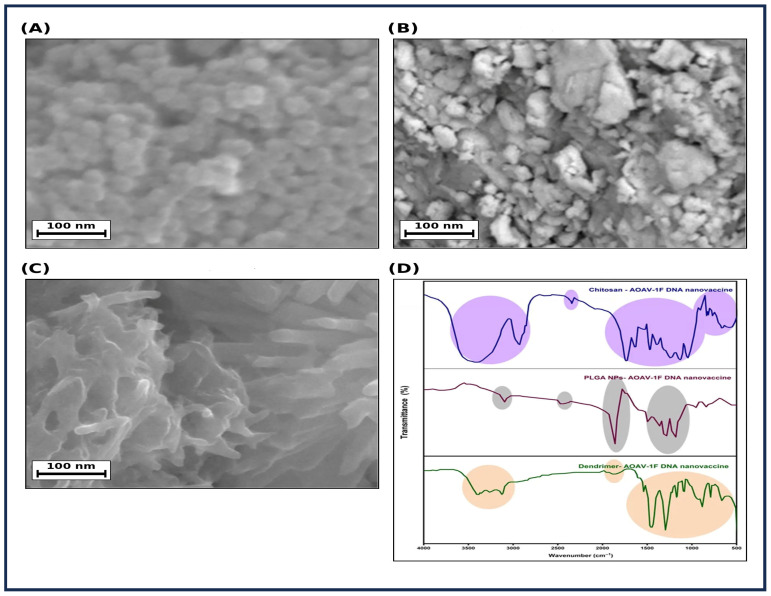
Morphological characterization and FTIR analysis of pDNA-F-loaded nanoparticle formulations. (**A**–**C**): Scanning electron microscopy (SEM) images of pDNA-F-loaded nanoparticle formulations: (**A**) Cs-NPs-pDNA-F, (**B**) PLGA-NPs-pDNA-F, and (**C**) PAMAM-Dendrimers-pDNA-F. Scale bars represent 100 nm. (**D**) Fourier transform infrared (FTIR) spectra of the corresponding formulations, demonstrating carrier-specific spectral features and changes consistent with pDNA-F association.

SEM analysis revealed distinct surface morphologies among the three nanoparticle formulations (Figure 2A–C). PLGA-NPs-pDNA-F consisted predominantly of discrete, relatively uniform, spherical nanoparticles with smooth surfaces and good dispersion, although minor aggregation was observed. The estimated particle diameters ranged from 50 to 100 nm. In contrast, Cs-NPs-pDNA-F exhibited a markedly different morphology, characterized by heterogeneous, irregularly shaped particles with rough surface texture. Extensive aggregation was evident, with the formation of larger micron-sized clusters composed of smaller submicron and nanoscale primary particles. The overall structure appeared less defined and more crystalline or flake-like compared with PLGA-NPs-pDNA-F. PAMAM-Dendrimers pDNA-F displayed a unique morphology, characterized by a highly porous and interconnected three-dimensional network. Rather than discrete nanoparticles, the formulation exhibited elongated, rod-like, or fibrous structures fused to form a complex web-like architecture.

#### 3.2.3. Fourier Transform Infrared (FTIR) Analysis

FTIR spectra confirmed the successful formation of pDNA-F-loaded nanoparticles using three different nanocarriers (Chitosan, PLGA, and PAMAM-Dendrimers) and revealed carrier-specific spectral features consistent with DNA association (Figure 2D). Key spectral changes, including alterations in amine- and amide-associated bands for chitosan and PAMAM formulations, support electrostatic interactions with the DNA phosphate backbone, while attenuation or masking of DNA-related signals in PLGA formulations is consistent with polymeric encapsulation. Complete peak assignments and detailed spectral interpretations for all formulations are provided in Supplementary Table S1. Collectively, these findings confirm successful nanoparticle formation with preserved carrier-specific signatures and evidence of pDNA-F association.

#### 3.2.4. Entrapment Efficiency (EE%) and Loading Capacity (LC%)

The entrapment efficiency (EE%) and loading capacity (LC%) of pDNA-F varied among the three nanoparticle formulations. PAMAM-Dendrimers-pDNA-F exhibited the highest EE% (95.2 ± 1.8%), indicating highly efficient complexation/entrapment of pDNA-F during nanoparticle formation. This value was higher than those obtained for the Cs-NPs-pDNA-F (82.53 ± 1.2%) and PLGA-NPs-pDNA-F (80.5 ± 2.1%). In contrast, PAMAM-Dendrimers-pDNA-F displayed the lowest LC% (4.8 ± 0.6%), reflecting the relatively low proportion of pDNA-F mass relative to the total nanoparticle mass. Cs-NPs-pDNA-F achieved the highest LC% (18.2 ± 2.5%), suggesting a greater capacity of the chitosan matrix to accommodate pDNA-F. PLGA-NPs-pDNA-F showed an intermediate LC% (12.78 ± 1.5%). Overall, an inverse trend was observed between EE% and LC% across the formulations, whereby PAMAM-Dendrimers-pDNA-F achieved maximal entrapment efficiency but minimal loading capacity, whereas Cs-NPs-pDNA-F exhibited the highest loading capacity with slightly lower, though still high, entrapment efficiency.

It should be noted that these EE% and LC% values are derived from an acid-extraction protocol that was not independently validated for complete DNA recovery or integrity. Accordingly, these values should be interpreted as comparative estimates under standardized conditions rather than absolute measures of encapsulated DNA quantity.

#### 3.2.5. In Vitro Release of pDNA-F DNA from Nanoparticles

**Figure 3:**
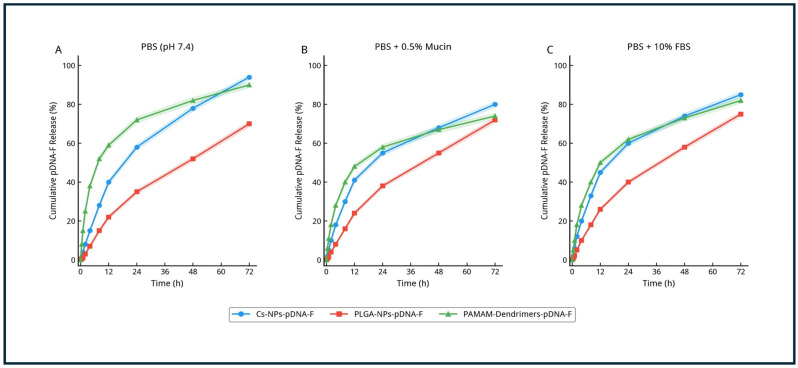
In vitro cumulative release profiles of pDNA-F from nanoparticle formulations under different physiological conditions. In vitro cumulative release of pDNA-F from Cs-NPs-pDNA-F, PLGA-NPs-pDNA-F, and PAMAM-Dendrimers-pDNA-F over 72 h under (**A**) PBS (pH 7.4), (**B**) PBS supplemented with 0.5% (*w*/*v*) mucin, and (**C**) PBS supplemented with 10% (*v*/*v*) fetal bovine serum (FBS), at 37 °C. The results demonstrate formulation-dependent and environment-responsive release kinetics. Data are presented as mean ± SD (n = 3).

To better approximate the extracellular environments encountered following administration, the in vitro release behavior of pDNA-F loaded nanoparticles was evaluated in three media: PBS (pH 7.4) as a baseline condition, PBS supplemented with 0.5% (*w*/*v*) mucin to simulate the mucosal environment, and PBS supplemented with 10% (*v*/*v*) FBS to simulate a protein-rich biological milieu (Figure 3).

Under PBS conditions, all three formulations exhibited time-dependent release profiles consistent with their carrier-specific DNA association mechanisms. PLGA-NPs-pDNA-F demonstrated the most controlled and sustained release, reaching a cumulative release of approximately 70% at 72 h, reflecting the sustained diffusion-controlled kinetics characteristic of matrix-encapsulated systems. PAMAM-Dendrimers-pDNA-F exhibited the most pronounced early release phase, reaching approximately 59% by 12 h and approximately 90% by 72 h, consistent with the reversible electrostatic nature of dendriplex formation. Cs-NPs-pDNA-F showed an intermediate-to-high cumulative release, reaching approximately 94% at 72 h, driven by polymer swelling and hydration.

In mucin-containing medium, the release profiles of all three formulations were altered relative to PBS, with the most pronounced changes observed for the cationic carriers. PAMAM-Dendrimers-pDNA-F and Cs-NPs-pDNA-F showed a reduction in cumulative release compared with their respective PBS profiles, with values of approximately 74% and 80% at 72 h, respectively. This behavior is consistent with strong electrostatic interactions between the positively charged carrier surfaces and the negatively charged sialic acid residues of the mucin glycoprotein network, which can retard nanoparticle diffusion and interfere with DNA release kinetics. In contrast, PLGA-NPs-pDNA-F maintained a comparatively controlled and stable release profile in mucin-containing medium (~72% at 72 h), with minimal deviation from its PBS profile, reflecting its lower electrostatic susceptibility to mucin interaction.

Under serum-containing conditions, all formulations exhibited further changes in release behavior compared with PBS. Cs-NPs-pDNA-F showed the greatest perturbation, with a cumulative release of approximately 85% at 72 h and a more variable early-phase profile, consistent with protein-mediated destabilization of its loosely associated matrix. PAMAM-Dendrimers-pDNA-F similarly showed modified kinetics (~82% at 72 h) relative to PBS, reflecting the formation of a serum protein corona on the cationic surface that may partially compete with or displace electrostatically bound pDNA-F. PLGA-NPs-pDNA-F demonstrated the most stable behavior in serum (~75% at 72 h), with the smallest deviation across all tested conditions. Collectively, these data demonstrate that PBS-only release testing does not fully capture the environmental sensitivity of these formulations, and that the cationic carriers are more susceptible to condition-dependent perturbation than the anionic PLGA system.

#### 3.2.6. In Vitro Stability of pDNA-F–Loaded Nanoparticles Under DNase I and Serum Conditions

The stability of the three pDNA-F nanoparticle formulations was further evaluated under nuclease-containing and serum-containing conditions to better reflect biologically relevant extracellular environments (Table 3). In the DNase I protection assay, naked pDNA-F underwent rapid enzymatic degradation, with a marked reduction in band intensity after 30 min and near-complete loss of the intact DNA band by 60 min. In contrast, nanoparticle-associated pDNA-F showed clear protection against nuclease digestion, although the degree of protection varied among carriers.

PLGA-NPs-pDNA-F and PAMAM-Dendrimers-pDNA-F exhibited the strongest protection, with clear residual intact DNA bands still detectable after DNase I treatment, indicating efficient shielding of the pDNA cargo against enzymatic attack. Cs-NPs-pDNA-F also protected pDNA-F relative to naked DNA, but the recovered DNA band was weaker, particularly at the longer incubation period, suggesting comparatively lower nuclease resistance. This reduced stability is consistent with the less compact and more heterogeneous morphology observed for the chitosan formulation.

In the serum stability assay, all nanoparticle formulations retained higher levels of intact pDNA-F than naked DNA, but marked formulation-dependent differences were evident. PLGA-NPs-pDNA-F showed the highest residual DNA integrity over time, maintaining approximately 92% intact pDNA-F at 4 h, 80% at 24 h, and 68% at 48 h. PAMAM-Dendrimers-pDNA-F also showed good early protection, with approximately 88% residual intact pDNA-F at 4 h, but this declined more substantially over time to approximately 65% at 24 h and 48% at 48 h, suggesting progressive destabilization in the presence of serum proteins. Cs-NPs-pDNA-F exhibited intermediate stability, maintaining approximately 78% intact pDNA-F at 4 h, 52% at 24 h, and 35% at 48 h. By contrast, naked pDNA-F was rapidly destabilized in serum, with only approximately 20% remaining intact at 24 h and less than 10% at 48 h.

Collectively, these data indicate that nanoparticle association substantially improves pDNA-F stability relative to naked DNA, but the magnitude and persistence of this protection are strongly carrier-dependent. Among the tested systems, PLGA-NPs-pDNA-F provided the most robust overall stability under both nuclease and serum conditions, whereas PAMAM-Dendrimers-pDNA-F provided strong nuclease protection but showed greater serum sensitivity, and Cs-NPs-pDNA-F exhibited only moderate stability.

### 3.3. Enhanced Post-Challenge Anamnestic Humoral Responses Following Vaccination with PAMAM-Dendrimers-pDNA-F

**Figure 4:**
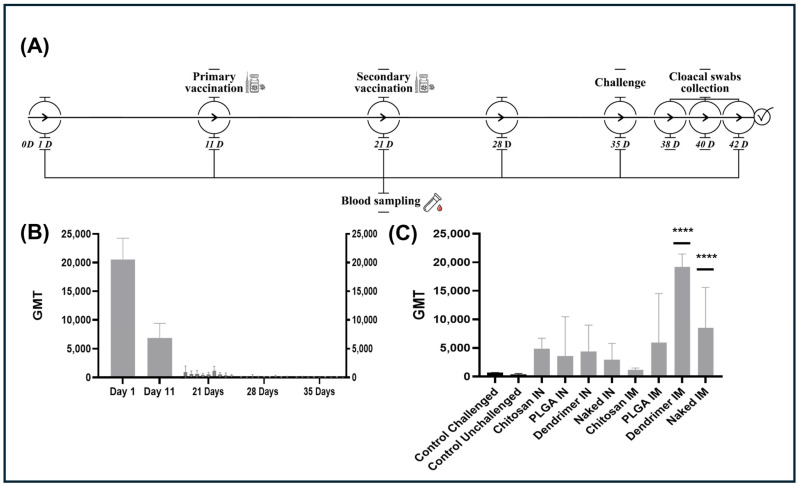
Experimental design and antibody response profile throughout the experiment. (**A**) Schematic timeline of the in vivo experimental design showing primary vaccination (day 11), booster vaccination (day 21), challenge (day 35), blood sampling points, and cloacal swab collection schedule. (**B**) Kinetics of geometric mean antibody titers (GMT) measured by ELISA in at different time points (days 1, 11, 21, 28, and 35). (**C**) Post-challenge geometric mean titers (GMT) of individual experimental groups. Data are presented as mean ± SD. Statistical significance is indicated as **** *p* < 0.0001.

Serological monitoring revealed a characteristic decline in maternally derived antibodies (MDA) during the first three weeks. At 1 and 11 days of age, prior to group allocation and vaccination (Figure 4A,B), the flock exhibited high baseline MDA levels (GMT = [20557] and [6836], respectively). By day 21, titers across all groups continued to wane as anticipated (Figure 4B). Although this sampling point occurred 10 days after the initial vaccination, the detected titers were consistent with the natural decay of maternal immunity and did not indicate a vaccine-induced humoral response. Notably, no active humoral response was detected at 28 or 35 days of age, and antibody titers in all experimental groups remained below the assay positivity threshold (Figure 4B).

After challenge, consistent with the induction of an anamnestic humoral response, antibody titers increased dramatically in the challenged groups, with substantial differences observed between experimental groups (Figure 4C). Regarding intramuscular groups, the PAMAM-Dendrimers-pDNA-F group showed the highest post-challenge GMT, followed by naked pDNA-F and PLGA-NPs-pDNA-F groups, whereas the Cs-NPs-pDNA-F group exhibited the lowest GMT. Moderate GMTs were observed in intranasal groups. The non-vaccinated-challenged control group developed only low antibody titers, while the non-vaccinated-unchallenged control group remained seronegative throughout the experimental period. Under standardized challenge conditions, these differences in post-challenge antibody responses may reflect variation in the priming efficiency of the different vaccine formulations.

### 3.4. PAMAM-Dendrimers-pDNA-F Exhibits Potent Protective Efficacy Against Virulent Challenge

**Figure 5:**
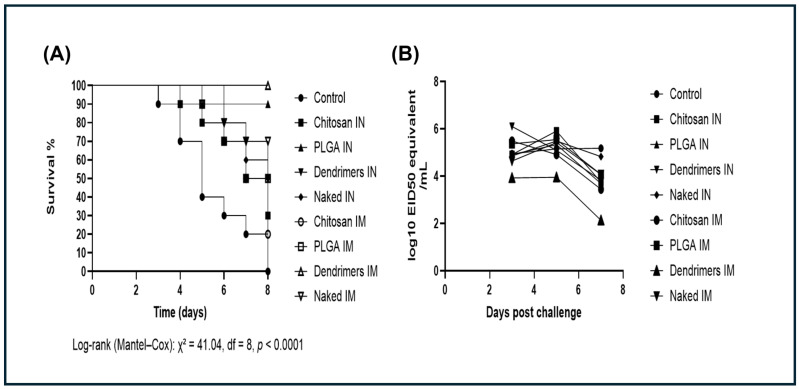
Survival outcomes and viral shedding following Virulent AOAV-1 Challenge. (**A**) Kaplan–Meier survival curves of chickens immunized with Cs-NPs-pDNA-F, PLGA-NPs-pDNA-F, and PAMAM-Dendrimers-pDNA-F via intranasal (IN) or intramuscular (IM) routes, compared with naked pDNA-F and non-vaccinated challenged controls, monitored for 8 days post-challenge (dpc). Survival differences were analyzed using the log-rank (Mantel–Cox) test (χ^2^ = 41.04, df = 8, *p* < 0.0001). (**B**) Viral shedding levels quantified as log_10_ EID_50_ equivalents in cloacal swabs at 3, 5, and 7 dpc. The data represent group-level measurements.

The protective efficacy of the different pDNA-F formulations against the virulent AOAV-1 challenge is illustrated by the Kaplan–Meier survival curves (Figure 5A), with overall survival rates summarized in Table 1. As expected, the non-vaccinated, challenged control group exhibited 100% mortality by 8 dpc, with deaths began at 2 dpc, confirming the virulence of the challenge strain, while the non-vaccinated, unchallenged control group showed 100% survival throughout the observation period. PAMAM-Dendrimers-pDNA-F IM group showed complete protection, with 100% survival throughout the observation period. PLGA-NPs-pDNA-F IN group showed 90% survival, with a single mortality recorded at 6 days post-challenge (dpc). Intermediate protection was observed in naked pDNA-F IM (70% survival) and naked pDNA-F IN (50% survival) groups. PLGA-NPs-pDNA-F IM and PAMAM-Dendrimers-pDNA-F IN groups each showed 50% survival. In contrast, Cs-NPs-pDNA-F showed the lowest survival rates, with 30% survival (IN) and 20% survival (IM).

Viral shedding was also quantified by RT-qPCR and expressed as log_10_ EID_50_ equivalents/mL using a standard curve generated from ten-fold serial dilutions of a quantified virus standard (Ct = −3.345 × log_10_[EID_50_] + 41.52; R^2^ = 0.9869), as indicated earlier [40]. The non-vaccinated, challenged control group exhibited sustained high-level viral RNA shedding (approximately 5.18 log_10_ EID_50_ equivalents/mL) throughout the study, confirming the robustness of the challenge model and supporting the subsequent reductions in viral shedding. The efficacy of the formulations in reducing viral RNA shedding varied according to both the nanoparticle carrier and the route of administration (Figure 5B). PAMAM-Dendrimers-pDNA-F IM group showed the most pronounced reduction in viral RNA load, maintaining the lowest level across all time points. Viral RNA levels declined from 3.92 log_10_ EID_50_ equivalents/mL at 3 dpc to 2.13 log_10_ EID_50_ equivalents/mL by 7 dpc. In contrast, PAMAM-Dendrimers-pDNA-F IN group exhibited higher viral RNA levels during the early phase (3 dpc), followed by a marked decline toward the end of the observation period. Cs-NPs-pDNA-F and PLGA-NPs-pDNA-F produced moderate reductions in viral RNA shedding compared with the control challenged group. Vaccination with naked pDNA-F (both IN and IM) provided limited control of viral RNA shedding, with values remaining above 4.0 log_10_ EID_50_ equivalents/mL at 7 dpc.

## 4. Discussion

Various strategies have been explored to enhance the efficacy of AOAV-1 DNA vaccines, including cytokine co-expression, electroporation-assisted delivery, codon optimization, and nanoparticle-based delivery systems [41,42]. However, studies employing polymeric nanocarriers have often relied on relatively high DNA doses and achieved variable levels of protection, raising concerns regarding their scalability and field applicability. Moreover, a direct comparative evaluation of different polymeric delivery systems under standardized, relatively low pDNA dosing remains lacking. Accordingly, the present study addresses this gap by providing a systematic comparison of nanocarriers for AOAV-1 DNA vaccine delivery, supporting their preliminary translational potential toward field-applicable vaccination strategies, pending further validation under field conditions.

In the present study, successful cytoplasmic expression of the F protein in DF-1 cells confirmed effective transcription and translation of the pDNA-F construct (Figure 1C). Western blot analysis further demonstrated the presence of the cleaved F-1 subunit (Figure 1D), providing evidence that the pDNA-F-expressed protein undergoes the authentic post-translational maturation necessary to elicit a protective immune response [43,44].

Nevertheless, the efficacy of a DNA vaccine is determined not only by transgene competence but also by the efficiency with which the genetic cargo is protected, internalized, and presented to the host immune system [45]. Therefore, we utilized three nanoparticle-based delivery systems: Chitosan, PLGA, and PAMAM Dendrimers, to overcome physiological obstacles that hinder the efficacy of pDNA in its naked form, to enhance the efficacy of the pDNA-F vaccine candidate. By comparing these formulations with naked pDNA-F administered via both intramuscular and intranasal routes, we aim to understand how vaccine design, nanocarrier properties, and delivery strategy collectively influence immune priming capacity and protective efficacy.

The efficacy of the delivery systems is fundamentally governed by the physicochemical properties of the carrier, specifically its size, size distribution, and surface charge, which dictate stability, cellular interactions, and immunogenicity [46]. All three formulations exhibit sizes within the optimal range (100–300 nm) for uptake by antigen-presenting cells (APCs) [47]. Measuring 120 ± 10.2 nm, PAMAM-Dendrimers were the smallest across all formulations, providing a distinct advantage in cellular internalization and tissue penetration [48]. Furthermore, the low PDI values (0.24) across all systems confirm a monodisperse population, which is crucial for reproducible in vivo performance [49]. Moreover, PAMAM-Dendrimers showed the greatest homogeneity, exhibiting the lowest PDI (0.16 ± 0.03). Surface charge, indicated by Zeta Potential, is a critical determinant of colloidal stability and cellular interaction. Both PAMAM-Dendrimers (+45.3 ± 3.5 mV) and Chitosan (+32.5 ± 2.8 mV) formulations were highly cationic, ensuring both high colloidal stability and a strong electrostatic affinity for the negatively charged host cell membranes, which are critical for enhancing cellular adhesion and subsequent endosomal uptake of the DNA payload [27,50]. In contrast, the PLGA formulation demonstrated a negative Zeta Potential of −21.8 ± 1.9 mV. This anionic characteristic is primarily attributed to the terminal carboxylic acid groups on the PLGA polyester backbone. While this charge provides moderate colloidal stability, it is a well-documented limitation of PLGA systems, as it may reduce initial cellular uptake efficiency due to electrostatic repulsion from the negatively charged sialic acid residues on host cell membranes [51].

Morphological assessment by SEM (Figure 2A–C) revealed that PAMAM-Dendrimers-pDNA-F exhibited an interconnected, web-like network characteristic of high-generation dendriplexes [52], which efficiently condenses the DNA into a compact state, shielding it from enzymatic degradation while maximizing the surface area available for membrane interaction. PLGA-NPs-pDNA-F was observed as spherical nanoparticles with smooth surfaces, suggesting that DNA is predominantly entrapped within the hydrophobic core rather than adsorbed to the surface. Cs-NPs-pDNA-F exhibited irregular, aggregated structures rather than the uniform spheres observed in PLGA.

Notably, these observations highlight the importance of interpreting nanoparticle characteristics in the context of measurement conditions. While DLS reflects hydrodynamic behavior in aqueous suspension, SEM represents the morphology of dried materials following sample preparation. Accordingly, the aggregated morphology observed for chitosan likely reflects enhanced interparticle interactions during drying, whereas the network-like structure of PAMAM is consistent with dehydration-induced structural reorganization of soft electrostatically assembled dendriplexes. SEM was used here primarily to illustrate comparative morphology rather than to directly validate the hydrodynamic size measured by DLS. Importantly, the aggregation behavior observed in chitosan-based systems may have biological implications, as it can affect dispersion stability, tissue penetration, and cellular uptake, thereby contributing to the reduced in vivo performance observed for this formulation.

The FTIR spectra (Figure 2D) validated the successful preparation of all three formulations and revealed that chitosan and PAMAM dendrimers primarily electrostatically complexed pDNA-F, whereas PLGA nanoparticles predominantly physically encapsulated pDNA-F.

The observed inverse relationship between encapsulation efficiency (EE%) and loading capacity (LC%) among the three formulations was a critical finding. For PAMAM-Dendrimers, while their structural precision facilitated the highest encapsulation efficiency (95.2 ± 1.8%), it inherently limited the total mass incorporation, resulting in the lowest loading capacity (4.8 ± 0.6%) of the three systems. For PLGA, while it achieved a substantial loading capacity, it exhibited the lowest encapsulation efficiency (80.5 ± 2.1%). This finding is a frequent challenge in emulsion-based methods, where water-soluble DNA can escape into the external aqueous phase [53,54]. Regarding Chitosan, its less-structured, porous matrix achieved the highest loading capacity (18.2 ± 2.5%) among all tested systems, suggesting that the natural polysaccharide’s architecture can accommodate a greater DNA payload mass than that of synthetic polymers.

The extended-release studies under mucin and serum-containing conditions revealed environment-dependent modulation of DNA release that was not apparent from PBS-based testing alone. The respiratory mucosa, which is the primary target of the intranasal route, presents a viscoelastic gel-like barrier composed predominantly of mucin glycoproteins with abundant negatively charged sialic acid and sulfate residues [55]. These residues readily interact with positively charged nanoparticles, promoting mucoadhesive interactions that restrict nanoparticle diffusion and reduce the effective availability of released DNA at the epithelial interface. The significant perturbation observed for PAMAM-Dendrimers-pDNA-F and Cs-NPs-pDNA-F in mucin-containing medium is therefore not unexpected and is mechanistically consistent with the mucus-trapping phenomenon invoked to explain the attenuated intranasal performance of these formulations in vivo. This reduction does not reflect improved sustained release, but rather indicates physical retention within the mucus network, which may limit effective delivery to underlying target cells. By contrast, the relatively stable release profile of PLGA-NPs-pDNA-F in mucin medium directly supports its suitability for intranasal delivery, aligning with the superior in vivo intranasal protection observed with this formulation (90% survival) [56].

The serum-containing release assay further demonstrated that the protein-rich environment encountered following intramuscular injection can also influence nanoparticle behavior. Serum proteins, including albumin, immunoglobulins, and fibrinogen, can adsorb onto cationic surfaces to form a protein corona, which may alter particle size, surface charge, colloidal stability, and cargo-release kinetics [57,58,59]. The greater sensitivity of PAMAM-Dendrimers-pDNA-F and Cs-NPs-pDNA-F to serum conditions is consistent with their high cationic charge, whereas the comparatively lower sensitivity of PLGA-NPs-pDNA-F may reflect the partial shielding effect of its PVA stabilizer layer and lower surface charge magnitude. Despite this, the superior intramuscular performance of PAMAM-Dendrimers-pDNA-F in vivo (100% survival) suggests that its modified kinetics in serum did not prevent sufficient DNA delivery to target cells, possibly because the early burst release facilitated rapid local availability of pDNA-F and efficient uptake by muscle cells before significant protein-mediated interference could occur.

Collectively, these findings demonstrate that nanoparticle performance is highly dependent on the interaction between carrier properties and the biological environment, and that release behavior under physiologically relevant conditions provides a more accurate framework for interpreting in vivo vaccine efficacy.

The in vitro stability assays confirmed that nanoparticle encapsulation substantially improved pDNA-F resistance to DNase I degradation and serum-mediated destabilization compared with naked pDNA-F, which was rapidly degraded with near-complete loss of detectable intact DNA under both conditions, highlighting the critical role of polymeric carriers in protecting the DNA payload before cellular delivery [60]. Among the tested formulations, PLGA-NPs-pDNA-F demonstrated the strongest overall stability, consistent with the protective effect of physical encapsulation within a polymeric matrix that limits enzyme access and improves cargo persistence in biological fluids [54]. PAMAM-Dendrimers-pDNA-F also showed strong DNase protection, likely due to tight electrostatic condensation of pDNA by the highly cationic dendrimer surface, although its comparatively lower serum stability may reflect protein corona formation and competitive interactions with serum components [52,61]. In contrast, the more moderate stability of Cs-NPs-pDNA-F may be attributable to its heterogeneous and aggregated structure, which likely allows greater DNA exposure and weaker protection in complex biological media [62]. Collectively, these findings indicate that nanoparticle-mediated DNA protection is strongly formulation-dependent and likely contributes to the route-specific biological performance observed in vivo.

The in vivo performance of the naked pDNA-F groups served as a baseline for evaluating whether nanoparticle use enhances or hinders candidate vaccine efficacy. Consistent with the other formulations in the study, post-vaccination ELISA titers remained below detection limits (Figure 4B), a characteristic frequently observed with DNA vaccines that prioritize cellular immunity and immunological memory [63,64]. The formulations effectively ‘primed’ the avian immune system. The impact of this priming was most evident following the challenge. The naked pDNA-F IM group exhibited a pronounced anamnestic response, recording the second-highest post-challenge antibody titers among all experimental groups, surpassed only by the PAMAM-Dendrimers-pDNA-F IM group (Figure 4C). Naked pDNA-F provided 70% survival via the intramuscular (IM) route and 50% survival via the intranasal (IN) route, consistent with earlier reports of partial protection by naked DNA vaccines against Newcastle disease in chickens [65,66]. The moderate survival observed in pDNA-F groups suggested that, at the concentration used, sufficient pDNA-F reached the myocytes or local APCs before being completely degraded by extracellular enzymes. However, the instability of naked DNA is well documented, and without a protective carrier, the genetic payload is highly susceptible to rapid enzymatic degradation by interstitial DNases. This hypothesis leads to suboptimal gene expression and failure to achieve the protective efficacy (≥90%) required [27].

PAMAM-Dendrimers-pDNA-F IM group exhibited a distinct anamnestic response, with antibody titers surging post-challenge faster and more vigorously than in other groups, suggesting it is a potent candidate (Figure 4C). This rapid reactivation of memory cells translated into superior clinical outcomes, including the highest survival rate (100%) and a profound reduction in viral RNA shedding (approximately a 1000-fold decrease in log_10_ EID_50_ equivalents/mL (Figure 5B), Importantly, this reduction extends the formulation’s significance beyond individual protection, as reduced viral shedding limits environmental contamination and transmission within the flock. Uncontrolled viral shedding, even in apparently healthy birds, sustains silent circulation and complicates disease control in endemic settings. Therefore, reducing viral shedding is essential to interrupt transmission cycles and improve farm-level biosecurity, particularly in high-density poultry production systems [67]. In this context, genotype-matched vaccines have been shown to reduce viral shedding more effectively, highlighting the importance of antigenic matching in NDV control [68]. Such matching can be more readily achieved using molecular-based vaccine platforms due to their adaptability to circulating strains.

However, the efficacy was notably attenuated via the intranasal (IN) route. The high cationic charge (+45.3 mV) of the dendriplexes likely facilitated electrostatic entrapment within the respiratory mucus. The strong attraction between the positive terminal amines of the dendrimer and the negatively charged sialic acid residues of the mucin glycoproteins creates a “mucus trap” [69,70], which significantly hinders the diffusion of the polyplexes toward the underlying target epithelium [71]. This immobilization is a well-documented phenomenon in which cationic nanoparticles become physically and electrostatically tethered to the mucin fiber network, preventing them from reaching the mucosal-associated lymphoid tissues (MALT) [72]. As a result, while surface charge is a primary driver for cellular uptake once at the cell membrane, it acts as a significant barrier during the initial mucosal transit. This observation highlights the importance of route-dependent design considerations, as physicochemical properties that enhance cellular uptake in systemic tissues may hinder effective transport across mucosal barriers. While surface modifications such as PEGylation or charge shielding could improve mucus penetration, these were not applied in the present study in order to evaluate the intrinsic behavior of the carrier systems.

The in vivo performance of PLGA-NPs-pDNA-F provided a compelling contrast between administration routes, with a clear difference in survival rate: the IN group achieved 90% survival, a level of protection significantly higher than that of the IM group, which showed only 50% survival. Unlike the cationic dendrimers that are susceptible to “mucus trapping,” the anionic PLGA particles likely experienced less electrostatic interference with the respiratory mucus, allowing for better diffusion and interaction with the Nasal-Associated Lymphoid Tissue (NALT) [71,72]. However, an anionic charge, which is advantageous for mucosal delivery, may limit uptake in muscle tissue [62]. The PLGA groups exhibited a slight anamnestic response post-challenge, with very low post-challenge GMTs compared with those of the dendrimer–IM group (Figure 4C). Despite this very low level, the high survival rate, especially in the intranasal group, may suggest a potential critical contribution of mucosal and cell-mediated immunity, although these mechanisms were not directly evaluated in the present study [73,74].

Cs-NPs-pDNA-F demonstrated the lowest performance in the animal trial. Although the onset of mortality was slightly delayed compared to the non-vaccinated, challenged control group (Figure 5A), the overall survival rate was very low. The irregular aggregation observed in the SEM analysis may have reduced the cellular uptake of the DNA. Larger clusters are less efficient at penetrating tissues or being internalized by APCs than the smaller, discrete particles in the PAMAM-Dendrimers (120 nm) or PLGA (255 nm) groups, contributing to the lowest overall performance [75]. Intramuscularly, the moderate stability of the chitosan formulation under serum conditions, together with its susceptibility to protein-mediated destabilization, may have promoted premature extracellular release and partial degradation of pDNA-F prior to efficient cellular internalization [76]. Intranasally, chitosan’s strong cationic charge and muco-adhesion likely tethered the particles to mucin glycoproteins in the respiratory mucus, preventing the DNA from diffusing toward the target NALT [69,70]. Notably, chitosan nanoparticles were evaluated in their unmodified form to enable a baseline characterization of this widely used carrier platform. While surface modification strategies, such as PEGylation, thiolation, or the incorporation of muco-penetrating coatings, are known to reduce mucoadhesion and improve mucus penetration, these approaches were not applied in the present study in order to preserve a direct comparison of the intrinsic physicochemical behavior of the carrier systems across administration routes.

Although cell-mediated and mucosal immune responses were not directly evaluated, the observed protection despite undetectable pre-challenge antibody responses suggests a potential contribution of non-humoral immune mechanisms. Consistent with this, protection against NDV has been reported following DNA vaccination despite the absence of detectable neutralizing antibodies, supporting a possible role for cell-mediated immunity in plasmid DNA-induced protection [41]. These findings emphasize that protective efficacy, as reflected in survival and reduced viral RNA shedding, does not necessarily correlate with systemic IgY levels measured by ELISA alone; rather, it may reflect a coordinated immune response involving cellular immunity and rapid post-challenge humoral expansion. In this context, DNA vaccines are recognized as effective priming platforms, inducing immune memory rather than strong immediate humoral responses and enabling rapid anamnestic responses upon pathogen exposure. This property reinforces their utility in heterologous prime–boost strategies, where DNA priming can be effectively applied even in the presence of maternally derived antibodies, followed by live or viral vector vaccines to enhance protective immunity.

Comparison of naked pDNA-F with nanoparticle-based formulations reveals a distinct carrier-dependent impact on the vaccine performance. The use of PAMAM-Dendrimers successfully elevated IM protection from 70% to 100%, while PLGA elevated IN protection from 50% to 90%. This finding confirms that selecting an appropriate carrier system, along with the appropriate route of administration, is essential for achieving optimal DNA vaccine potency. Conversely, Cs-NPs-pDNA-F was less effective than naked DNA, with survival rates dropping to 20–30%. This data suggests that an inappropriate carrier can be more detrimental than providing no carrier at all. The naked pDNA-F, being smaller and naturally anionic, likely diffused through the respiratory mucus or muscle tissue more effectively than the large, “trapped” chitosan clusters formed under the present formulation conditions.

This study was designed to comparatively evaluate formulation-dependent biological performance under controlled experimental conditions, rather than to establish field-ready vaccination strategies; accordingly, any implications for field application remain preliminary and require validation under commercial conditions. Within this context, PAMAM-Dendrimers-pDNA-F demonstrated superior protective efficacy, reflecting its high delivery efficiency and positioning it as the most biologically robust formulation evaluated in this study. While considerations related to manufacturing complexity and scalability may limit its immediate application in mass vaccination programs, its strong performance supports continued investigation, particularly for targeted or high-value applications. In contrast, the PLGA-NPs-pDNA-F intranasal formulation offers practical advantages for large-scale field deployment, including compatibility with mass, needle-free administration, and rapid flock-wide immunization. Collectively, these findings highlight the importance of balancing immunological performance with practical considerations for effective implementation in poultry production systems.

Given the comparative and multi-factorial design of the present study, several limitations should be acknowledged to facilitate accurate interpretation of the findings.

First, several methodological considerations may have influenced the physicochemical characterization of the formulations. The determination of entrapment efficiency (EE%) and loading capacity (LC%) relied on acid extraction using 0.1 N HCl followed by spectrophotometric quantification, a method that was not independently validated for extraction completeness or preservation of DNA integrity across the different polymeric systems. In particular, the susceptibility of PLGA to hydrolytic degradation under acidic conditions may have led to partial DNA loss or degradation, potentially resulting in underestimation of the true encapsulated pDNA-F content. Future work should include spike-recovery controls and agarose gel-based integrity verification to confirm that the extraction protocol yields quantitatively representative and structurally intact DNA prior to spectrophotometric measurement. In addition, particle size and morphology were assessed using dynamic light scattering (DLS) and scanning electron microscopy (SEM), which represent fundamentally different physical states (hydrated versus dried conditions), likely contributing to the observed discrepancies between size distribution profiles and morphological appearance, Future studies employing techniques such as cryo-transmission electron microscopy (cryo-TEM) or nanoparticle tracking analysis (NTA) would provide more accurate characterization of nanoparticle structure under hydrated conditions. 

Second, the experimental design incorporated multiple formulations, administration routes, and outcome measures within a single study framework. While this enabled a comprehensive comparative evaluation, it inherently limited the depth of optimization achievable for each individual variable. No surface modifications intended to enhance mucus penetration (e.g., PEGylation or charge shielding) were applied to the nanoparticle formulations, which may have limited the performance of cationic carriers after intranasal administration. Future studies should incorporate such strategies to optimize nanocarrier design for mucosal delivery. In addition, normalization of treatment groups based on an equal pDNA-F dose (40 µg), rather than an equivalent carrier mass, resulted in differences in nanoparticle quantities across formulations due to their distinct loading capacities. Consequently, observed differences in protective efficacy may partly reflect carrier-related effects, such as local inflammatory responses or cytotoxicity, rather than DNA delivery efficiency alone. Furthermore, although the administered DNA dose falls within the reported experimental range, it represents the lower end of commonly used concentrations [77,78,79,80], which may have contributed to the absence of detectable pre-challenge humoral responses.

Third, the biological and immunological evaluation of the vaccine candidates was limited in scope. The expressed F protein was C-terminally FLAG-tagged, and although the tag is located within the cytoplasmic tail and is unlikely to interfere with major extracellular neutralizing epitopes, its potential influence on higher-order structural conformation and antigen presentation cannot be entirely excluded. No direct assessment of cellular uptake or targeting (e.g., dendritic cell uptake) was performed, limiting mechanistic interpretation of nanoparticle-mediated delivery.

Fourth, the immunological assessment was primarily based on an NDV-F-specific indirect ELISA, without including functional assays such as serum neutralization or hemagglutination inhibition assays. Moreover, key components of protective immunity, particularly mucosal IgA responses and cell-mediated immunity (e.g., interferon-gamma production), were not evaluated. Future studies should incorporate these immunological endpoints, ideally using dedicated animal cohorts, to provide a more comprehensive understanding of the underlying protective mechanisms. In addition, longitudinal sampling of identified individuals and expanded sample sizes would enable a more accurate characterization of immune response kinetics and inter-individual variability following DNA vaccination. Furthermore, the absence of vector-only or carrier-only control groups limits the ability to fully distinguish antigen-specific immune responses from potential non-specific immunostimulatory effects of the delivery systems.

Finally, certain limitations related to virological assessment and outcome resolution should be considered. Viral shedding was assessed solely by cloacal swabs, which may not fully reflect respiratory tract replication, particularly after intranasal administration. Shedding analysis was based on pooled samples at each time point, precluding statistical comparisons and limiting the assessment of inter-individual variability. Furthermore, the study was restricted to a relatively short post-challenge observation period and did not include standardized clinical scoring systems or production-related performance parameters, such as body weight gain or feed conversion efficiency, which are important for evaluating field applicability. Future studies incorporating individual-level sampling, extended follow-up periods, and broader virological and clinical assessments would provide a more comprehensive evaluation of vaccine performance and its practical relevance under field conditions.

## Figures and Tables

**Table 1:** Experimental grouping based on nanoparticle carrier, administration route, and post-challenge survival.

Gr.	Formulation Administered	Carrier	Route	Challenge	Survival Rate
1	Non-vaccinated-challenged control	—	—	+	0%
2	Non-vaccinated-unchallenged control	—	—	-	100%
3	Cs-NPs-pDNA-F	Chitosan	Intranasal	+	30%
4	PLGA-NPs-pDNA-F	PLGA	Intranasal	+	90%
5	PAMAM-Dendrimers-pDNA-F	PAMAM-Dendrimers	Intranasal	+	50%
6	Naked pDNA-F	None	Intranasal	+	50%
7	Cs-NPs-pDNA-F	Chitosan	Intramuscular	+	20%
8	PLGA-NPs-pDNA-F	PLGA	Intramuscular	+	50%
9	PAMAM-Dendrimers-pDNA-F	PAMAM-Dendrimers	Intramuscular	+	100%
10	Naked pDNA-F	None	Intramuscular	+	70%

**Table 2:** Physicochemical characterization of pDNA-F-loaded nanoparticles measured by dynamic light scattering (DLS). Data are presented as mean ± SD (n = 3).

Formulation	Hydrodynamic Size (nm)	Polydispersity Index (PDI)	Zeta Potential (mV)
Cs-NPs-pDNA-F	210 ± 15.5	0.21 ± 0.04	+32.5 ± 2.8
PLGA-NPs-pDNA-F	255 ± 20.1	0.24 ± 0.05	−21.8 ± 1.9
PAMAM-Dendrimers-pDNA-F	120 ± 10.2	0.16 ± 0.03	+45.3 ± 3.5

**Table 3:** Comparative stability of pDNA-F–loaded nanoparticles under nuclease and serum conditions.

Formulation	DNase I (30 min)	DNase I (60 min)	DNase Protection Level	Residual Intact pDNA-F (in 10% FBS at 4 h) (%)	Residual Intact pDNA-F (in 10% FBS at 24 h) (%)	Residual Intact pDNA-F (in 10% FBS at 48 h) (%)	Colloidal Stability in Serum
Naked pDNA-F (control)	Faint /Markedly degraded band	No detectable intact band	None	55 ± 3	20 ± 2	8 ± 1	Poor
Cs-NPs-pDNA-F	Intact but weaker band	Weak residual band/partial degradation	Moderate	78 ± 4	52 ± 3	35 ± 3	Moderate; aggregation likely
PLGA-NPs-pDNA-F	Strong intact band	Clear residual intact band	High	92 ± 3	80 ± 4	68 ± 3	Good
PAMAM-Dendrimers-pDNA-F	Strong intact band	Moderate-to-strong residual band	High	88 ± 3	65 ± 4	48 ± 3	Moderate; protein corona likely

DNase I stability was assessed qualitatively by agarose gel electrophoresis after incubation with DNase I at 37 °C. Serum stability values are expected residual intact pDNA-F levels after incubation in PBS containing 10% FBS. Values are shown as expected mean ± SD for comparative interpretation.

## Data Availability

The original contributions presented in this study are included in the article. Further inquiries can be directed to the corresponding author.

## Supplementary Materials

The following supporting information can be downloaded at: https://www.mdpi.com/article/10.3390/v18050581/s1. Supplementary Methods S1. N/P Ratio Calculations. Table S1: FTIR peak assignments for Cs-NPs-pDNA-F, PLGA-NPs-pDNA-F, and PAMAM-Dendrimers-pDNA-F.

## Abbreviations

ND	Newcastle disease
AOAV-1	*Avian Orthoavulavirus 1*
F	Fusion
pDNA	Plasmid DNA
NPs	Nanoparticles
Cs	Chitosan
PLGA	Poly (lactic-co-glycolic acid)
PAMAM	Poly(amidoamine)
DLS	Dynamic light scattering
PDI	Polydispersity index
SEM	Scanning Electron Microscopy
FTIR	Fourier-transform infrared spectroscopy
IFA	Indirect immunofluorescence assay
WB	Western blot
ELISA	Enzyme-Linked Immunosorbent Assay
GMT	Geometric mean titer
RT-qPCR	Reverse Transcription quantitative Polymerase Chain Reaction
Ct	Cycle threshold
EID_50_	50% Egg Infectious Dose
Dpc	Day post challenge
