# Supplementary material for: Comparative Evaluation of Polymeric Nanocarriers for DNA Vaccine Delivery Against Avian Orthoavulavirus 1 in Chickens
Source: Viruses. 2026 May 21;18(5):581. doi: 10.3390/v18050581 (PMC13211407; doi:10.3390/v18050581)
Supplement: Supplementary file 1 [file viruses-18-00581-s001.zip › viruses-4177159-supplementary.pdf]

Supplementary Methods S1. N/P Ratio Calculations.

Cs-NPs-pDNA-F (N/P = 3:1): The N/P ratio for chitosan-based nanoparticles was defined as the molar ratio of the primary amine groups (–NH_2) of chitosan to the phosphate groups of the pDNA backbone. Chitosan (75% degree of deacetylation, medium molecular weight) provides approximately one accessible amine group per deacetylated glucosamine unit (molecular weight ≈ 161 Da). The phosphate group content of pDNA was calculated based on two phosphate groups per base pair, with an average molecular weight of 660 Da per base pair. The N/P ratio was optimized empirically at 3:1 to achieve complete DNA complexation while maintaining colloidal stability, as confirmed by gel retardation and zeta potential measurements.

PAMAM-Dendrimers-pDNA-F (N/P = 10:1): The N/P ratio for PAMAM dendriplex formation was defined as the molar ratio of the nitrogen atoms in the surface amine groups of the G5 PAMAM dendrimer to the phosphate groups of the pDNA backbone. G5 PAMAM dendrimers (molecular weight: 28,826 Da) carry 128 primary surface amine groups per molecule, yielding a nitrogen content of 128 mol N per mol dendrimer. The phosphate group content of pDNA was calculated as above (2 phosphate groups per base pair, average 660 Da per base pair). The N/P ratio was set to 10:1 based on prior optimization to ensure complete DNA condensation and formation of stable, compact dendriplexes.

Supplementary Table S1. FTIR peak assignments for Cs-NPs-pDNA-F, PLGA-NPs-pDNA-F, and PAMAM-Dendrimers-pDNA-F.

**Formulation**	**Wavenumber (cm^{-1})**	**Assignment**	**Interpretation**
Cs-NPs-pDNA-F	3600–3000	O–H / N–H stretching	Hydrogen bonding, hydrated structure
	1620–1650	Amide I (C=O stretch)	Chitosan backbone
	~1550	Amide II / N–H bending (shifted)	Electrostatic interaction between protonated amine groups and DNA phosphate backbone
	1150–1000	C–O–C / C–O stretching	Polysaccharide backbone
PLGA-NPs-pDNA-F	~1750	Ester C=O stretching	PLGA polymer matrix confirmed
	1250–1050	C–O stretching (broad)	PLGA backbone; masks DNA phosphate bands
	~1240 / ~1080	DNA phosphate bands (masked)	Physical encapsulation within PLGA core
PAMAM-Dendrimers-pDNA-F	~3290	N–H stretching	Dendrimer amine groups
	~1650	Amide I	Dendrimer internal amide bonds
	~1550	Amide II	Dendrimer internal structure
	1300–900	Complex fingerprint region	Overlapping dendrimer and DNA phosphate vibrations, consistent with dendrimer–DNA complex (dendriplex) formation
